# Selective modulation of the bone remodeling regulatory system through orthodontic tooth movement—a review

**DOI:** 10.3389/froh.2025.1472711

**Published:** 2025-03-06

**Authors:** Jan Christian Danz, Martin Degen

**Affiliations:** ^1^ Department of Orthodontics and Dentofacial Orthopedics, School of Dental Medicine ZMK, University of Bern, Bern, Switzerland; ^2^ Laboratory for Oral Molecular Biology, Department of Orthodontics and Dentofacial Orthopedics, University of Bern, Bern, Switzerland

**Keywords:** bone, orthodontic tooth movement, wound healing, osteoporosis, sclerostin, PTHrP, BMP, photobiomodulation

## Abstract

Little is known about how tissues mediate the ability to selectively form or resorb bone, as required during orthodontic tooth movement (OTM), facial growth, continued tooth eruption and for healing after fractures, maxillofacial surgical repositioning or implant dentistry. OTM has the unique ability to selectively cause apposition, resorption or a combination of both at the alveolar periosteal surface and therefore, provides an optimal process to study the regulation of bone physiology at a tissue level. Our aim was to elucidate the mechanisms and signaling pathways of the *bone remodeling regulatory system (BRRS)* as well as to investigate its clinical applications in osteoporosis treatment, orthopedic surgery, fracture management and orthodontic treatment. OTM is restricted to a specific range in which the BRRS permits remodeling; however, surpassing this limit may lead to bone dehiscence. Low-intensity pulsed ultrasound, vibration or photobiomodulation with low-level laser therapy have the potential to modify BRRS with the aim of reducing bone dehiscence and apical root resorption or accelerating OTM. Unloading of bone and periodontal compression promotes resorption via receptor activator of nuclear factor κB-ligand, monocyte chemotactic protein-1, parathyroid hormone-related protein (PTHrP), and suppression of anti-resorptive mediators. Furthermore, proinflammatory cytokines, such as interleukin-1 (IL-1), IL-6, IL-8, tumor necrosis factor-α, and prostaglandins exert a synergistic effect on bone resorption. While proinflammatory cytokines are associated with periodontal sequelae such as bone dehiscence and gingival recessions, they are not essential for OTM. Integrins mediate mechanotransduction by converting extracellular biomechanical signals into cellular responses leading to bone apposition. Active Wnt signaling allows β-catenin to translocate into the nucleus and to stimulate bone formation, consequently converging with integrin-mediated mechanotransductive signals. During OTM, periodontal fibroblasts secrete PTHrP, which inhibits sclerostin secretion in neighboring osteocytes via the PTH/PTHrP type 1 receptor interaction. The ensuing sclerostin-depleted region may enhance stem cell differentiation into osteoblasts and subperiosteal osteoid formation. OTM-mediated BRRS modulation suggests that administering sclerostin-inhibiting antibodies in combination with PTHrP may have a synergistic bone-inductive effect. This approach holds promise for enhancing osseous wound healing, treating osteoporosis, bone grafting and addressing orthodontic treatments that are linked to periodontal complications.

## 1 Introduction

Bone is not an inert structure, but rather a highly dynamic tissue that is constantly remodeled. To maintain a balance between bone formation by osteoblasts and resorption by osteoclasts, a bone remodeling regulatory system (BRRS) is required (1–4). The BRRS contributes significantly to the structural integrity of our skeletal system by allowing bones to specifically respond to mechanical stressors as well as to other biological components, such as the endocrine, immunological, and neurological systems (5). Any imbalance in bone remodeling can cause osteoporosis or osteopetrosis (6–10). The BRRS also has an endocrine function. Osteocytes synthesize and secrete fibroblast growth factor 23 (FGF23), which is known to act on distant organs. In the kidney, FGF23 inhibits 1α-hydroxylation of vitamin D and promotes phosphorus secretion, while in the parathyroid glands it leads to reduced secretion of PTH (8). Additionally, the BRRS enhances insulin synthesis in pancreatic β cells and increases glucose consumption in the peripheral tissue via osteocalcin in osteoblasts (11, 12).

Orthodontic tooth movement (OTM) requires the application of external forces to teeth, which elicits highly precise cellular responses that culminate in periodontal ligament (PDL) remodeling leading to bone resorption at the compression side of the moving tooth and bone apposition at the tension side. Indeed, the BRRS is selectively mediated by the PDL, a fibrous joint that suspends the root of each tooth in its alveolar bone socket. This mediation promotes resorption and apposition on both sides of the tooth root, which is necessary for successful OTM. Cells in the PDL play important roles in translating orthodontic stressors into biochemical signals that influence bone biology locally (13). The PDL is required for OTM since tooth movement does not happen when the ligament is partially absent, as observed in cases of ankylosis (14). However, it is still unclear how orthodontic forces precisely resorb or form bone to allow tooth movement, and how these remodeling processes are restricted, particularly at the alveolar cortical bone plate. The purpose of this review is to elucidate the role of the PDL in modulating bone biology locally, to decipher the involvement of parathyroid hormone-related protein (PTHrP) and sclerostin as important signaling molecules during OTM, and to propose strategies for avoiding OTM-related adverse effects.

## 2 Signaling of resorption in the periodontal pressure zone

**Figure 1:**
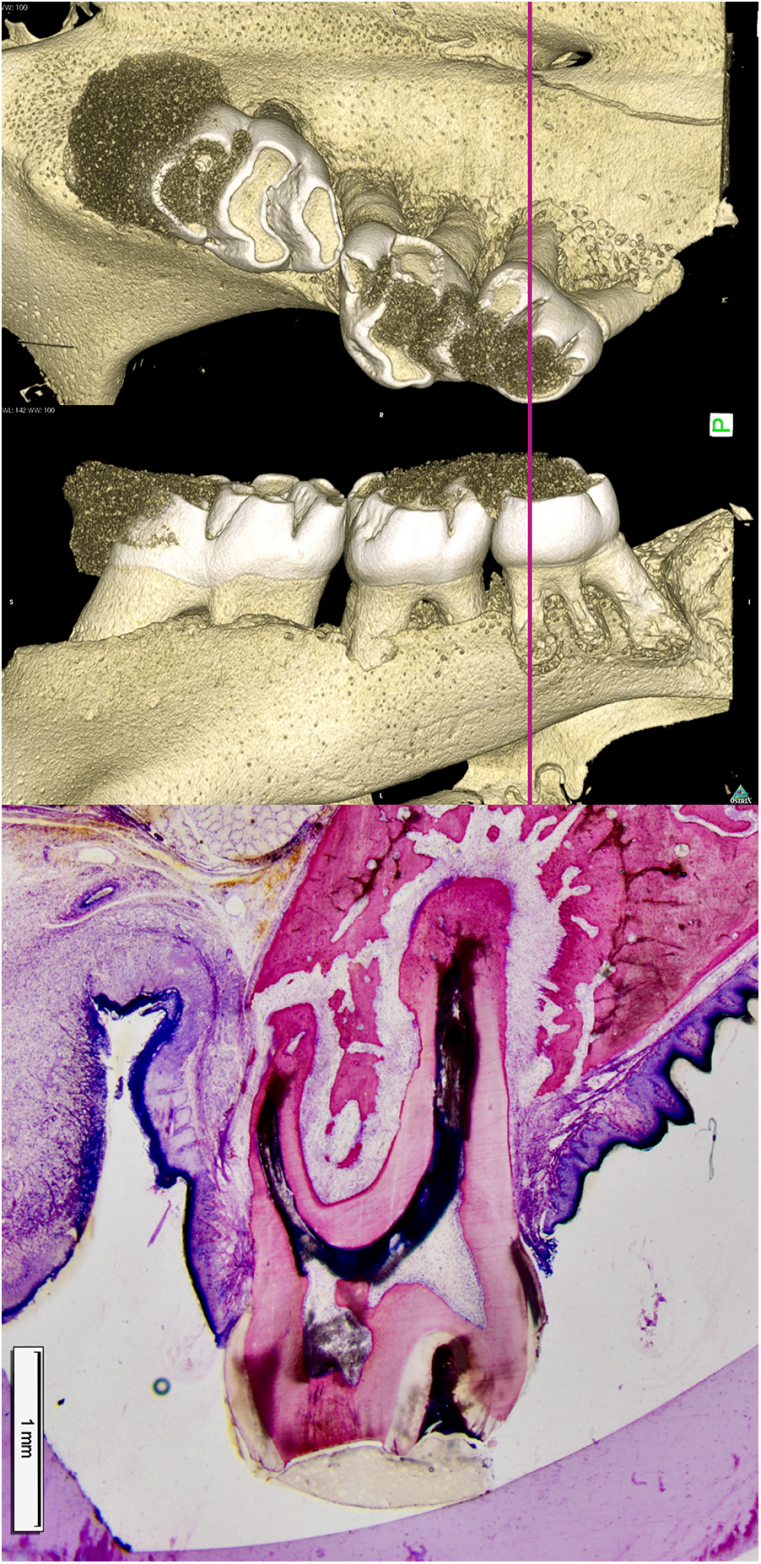
Bone resorption ahead of a root (pressure zone) and bone apposition in the periodontal tension zone. In a rat model for translational tooth movement, the second and third molars have been extensively displaced beyond the cortical surface of the alveolar bone. In the pressure area (left to the roots), resorption lacunae containing multinucleated osteoclasts are actively resorbing bone ahead of the root. The periodontal ligament (PDL) is wider in the tension area (right side of the roots), resulting in increased tooth mobility. Osteoblasts form bone in the tensile area to restore the PDL back to its normal width. A clear demarcation between older and younger bone is visible at the site where the alveolus was previously located, as the newly formed bone has not yet undergone complete remodeling (15).

A constant orthodontic force can cause continuous tooth movement by frontal resorption in the post-lag phase (15) (Figure 1). Osteoclasts are the only cell type capable of dissolving the bone matrix by secreting acids and specific proteases; thus, they play an essential role in bone remodeling in both health and disease. They are derived from hematopoietic stem cells in the bone marrow, which are released into the peripheral circulation as monocytes, cluster at the sites of bone resorption, and then differentiate into multinucleated osteoclasts (16). During physiological bone turnover, capillaries in the Haversian channels allow monocytes to travel throughout the body, including compact bone, where they develop into osteoclasts (1). For instance, during the initial 3 days of post-fracture wound healing, monocyte chemotactic protein-1 (MCP-1) is produced at the fracture site recruiting monocytes that differentiate into macrophages and osteoclasts (17).

The molecular and cellular mechanisms governing osteoclast differentiation have been widely studied. Macrophage colony-stimulating factor (M-CSF), expressed by fibroblasts, pericytes, and osteoblasts (18), and receptor activator of nuclear factor κB-ligand (RANKL) are critical factors for osteoclastogenesis. Although M-CSF is essential for the survival and proliferation of osteoclast precursor cells, RANKL, a member of the tumor necrosis factor superfamily, stimulates the development of precursor cells into osteoclasts and its levels are thought to correlate with the activation of osteoclast differentiation (16, 19). Nonetheless, full differentiation of myeloid precursor cells into osteoclasts and their survival are dependent on the combined activity of M-CSF and RANKL. RANKL exists in two forms: a membrane-bound protein and a soluble molecule (sRANKL) (20). Activated osteocytes and osteoblasts secrete sRANKL, which activates osteoclasts and induces bone resorption. It also promotes osteoclast cell differentiation by activating the RANK receptor on hematopoietic pre-osteoclastic cells (18, 21). Other transcription factors, including c-Fos, NF-κB, and nuclear factor-activated T-cells-1 also play relevant roles in modulating osteoclast differentiation (22, 23).

During OTM, compressed PDL fibroblasts upregulate RANKL, PTHrP, and MCP-1 while simultaneously downregulate osteoprotegerin (OPG) to mediate resorption at pressure zones (24–31).

### 2.1 RANKL/OPG

Osteoclast activity is primarily controlled by RANKL signaling, which can be inhibited by osteoblastic cell-secreted or serum OPG (20). As a result, the balance of RANKL and OPG at the PDL compression side contributes significantly to bone remodeling (32), with a higher RANKL/OPG ratio favoring RANKL-mediated osteoclastogenesis.

**Figure 2:**
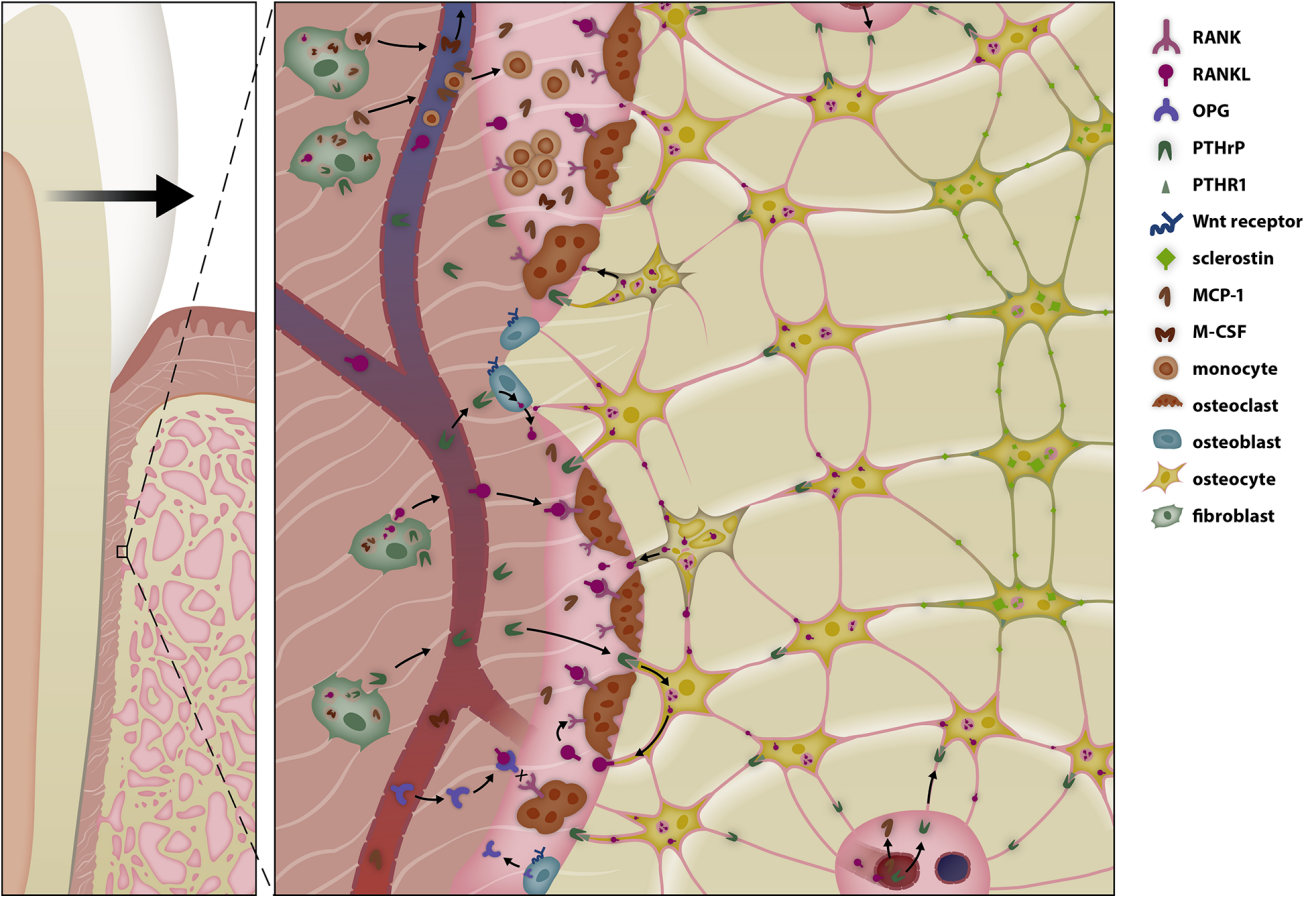
Selective modulation of the bone remodeling regulatory system (BRRS) for resorption by mechanotransduction in the late phase of orthodontic tooth movement (OTM). Periodontal fibroblasts initiate periodontal remodeling in the pressure area by increasing secretion of receptor activator of nuclear factor κB-ligand (RANKL) and macrophage colony-stimulating factor (M-CSF) and by reducing secretion of osteoprotegerin. Cytokines, prostaglandins, and growth factors play a pivotal role in the initial phase of tooth movement, enabling macrophages to remove necrotic tissue—especially after tipping movements. In the late phase of tooth movement, periodontal fibroblasts, the relaxation of periodontal fibers, and possibly the epithelial rests of Malassez modulate the BRRS so that bone resorption occurs in front of the root. Parathyroid hormone-related protein (PTHrP) activates surrounding cells of the osteoblastic lineage to release soluble RANKL (sRANKL), which together with the release of monocyte chemotactic protein-1 (MCP-1) increases osteoclastogenesis. Unloading of the PDL reduces the anabolic responses of osteoblasts in the pressure area, resulting in resorption along the entire root length during bodily tooth movement.

Indeed, human gingival crevicular fluid (GCF) samples were analyzed for sRANKL and OPG during OTM. Twenty-four hours after orthodontic force application, sRANKL levels increased significantly, while OPG levels remained lower in the GCF samples when compared to untreated teeth (33–35). PDL fibroblasts upregulate sRANKL while decreasing OPG levels *in vitro* in response to physiological orthodontic compressive forces, resulting in an elevated RANKL/OPG ratio, facilitating bone resorption for the root (29, 31). The kinetics of sRANKL upregulation revealed that its level peaked on day 2 and then dropped to control levels by day 4. Simultaneously, 48 h of OTM force application resulted in the greatest decrease in OPG levels, which only gradually returned to normal (29). Similar findings were reported in an *in vivo* OTM model in rats (36). Low force elicited a robust increase of sRANKL during the early phase of OTM, followed by a rapid normalization around the transition between early and late phase. The most substantial drop in OPG occurred in the early phase of OTM, and its levels reverted to baseline between days 6 and 58 of OTM treatment. As a result, the RANKL/OPG ratio initially increased in the early stages of OTM before stabilizing in the kate stages. This suggests that signaling mechanisms other than direct and continuous RANKL secretion are involved in the maintenance of pre-root bone resorption (Figure 2).

### 2.2 PTHrP

As a regulator of calcium and phosphorus levels, PTH plays a dual role in bone remodeling. PTH or PTHrP interaction with the PTH/PTHrP type 1 receptor (PTHR1) on osteocytes and osteoblasts increases bone turnover (37, 38), enhancing both apposition through decreased sclerostin production and enhanced osteoblast quantity and survival, as well as resorption via RANKL-mediated osteoclast activation.

PTH is clinically used in patients affected by osteoporosis and has been shown to stimulate bone catabolic or anabolic activity (7). This dual role depends on the presence of cofactors and circumstances of administration (37, 39). In contrast to continuous long-term exposure to PTH, which results in bone resorption, intermittent PTH application promotes bone growth (40). During tooth movement, the resorptive pathway is prominent due to persistent PTHR1 activation, which accelerates OTM. Under compressive conditions, PDL fibroblasts elevate PTHrP expression (28), activating osteoclasts and keeping resorption ahead of the root. In line with this, continuous, rather than intermittent, PTH administration in rats increases osteoclast numbers and speeds tooth displacement (41). PTHrP stimulates PTHR1 on osteoblasts and adjacent osteocytes, upregulating the resorption-promoting cytokines RANKL and M-CSF while downregulating OPG (8, 39, 42–44).

Furthermore, PTHR1 activation in osteocytes promotes bone mass through a low-density lipoprotein receptor-related protein 5 (LRP5)-dependent pathway that inhibits the Wingless (Wnt) antagonist sclerostin (39). The discovery of biallelic loss-of-function mutations in the *LRP5* gene in osteoporosis-pseudoglioma, an autosomal recessive disease characterized by loss of osteoblast function and thus low bone mass demonstrated the importance of the Wnt co receptor LRP5 in bone remodeling (45). In contrast, a gain-of-function LRP5 variant, LRP5_V171_, has been reported to cause an autosomal dominant syndrome characterized by osteoblast hyperactivity, high bone density with a thickened mandible and torus platinus (46). Recently, the DNA of seven patients with dental anomalies and oral extoses was sequenced and several variants of the *LRP5* genes were found (46, 47).

PTHrP plays a vital function in tooth eruption and movement. Primary failure of eruption is an autosomal dominant hereditary disease that causes pathological impairment of tooth eruption and mobility due to a heterozygous *PTHR1* mutation (48–50).

### 2.3 MCP-1

During tooth movement, MCP-1 is produced by human PDL fibroblasts in a force-dependent manner, increasing the number of osteoclasts on the alveolar bone and root surface (26). MCP-1 levels are also elevated in obesity, which has been shown to have a systemic effect on the BRRS by suppressing osteoblasts and osteocytes (51–53). MCP-1 along with RANKL, RANKL/OPG (54), and Dickkopf-1 (55), a soluble inhibitor of Wnt/β-catenin signaling, stimulate osteoclast development and activity. Aside from fat cells, osteoblasts express adiponectin and its receptors (56), indicating a direct influential link to bone metabolism. Therefore, there is compelling evidence that adipose tissue accelerates tooth movement (51, 57).

## 3 Role of inflammation in the periodontal pressure zone

Soon after the activation of orthodontic appliances, patients experience the initial symptoms of an inflammatory reaction, including pain, increased blood flow, and discomfort during chewing, which normally last a few days (58, 59). At the molecular level, mechanical force applied to periodontal cells triggers a biological response known as aseptic non-bacterial-induced inflammation. The PDL compression side is distinguished by changes in the vascular network, such as vasodilatation caused by distorted nerve endings and the release of vasoactive neurotransmitters, increased permeability, and vessel squeezing. Local hypoxia develops, followed by the formation of necrotic areas (60). Within 1–2 days of tooth movement in pressure zones, the altered microenvironment triggers the release of cytokines, prostaglandins, and growth factors (e.g., cyclooxygenase-2, IL-1, IL-6, IL-8, tumor necrosis factor-α), allowing macrophages to repair necrotic areas in the PDL. Elevated RANKL/OPG ratios (61–65), IL-1, IL-6, or IL-17, and tumor necrosis factor-α levels stimulate osteoclastogenesis and bone resorption in the PDL microenvironment (66, 67). The remodeling of the PDL is then initiated and supported by osteoclasts. However, because the compressed PDL precludes osteoclast differentiation, osteoclasts must be recruited from the surrounding alveolar bone using a mechanism called undermining resorption (66, 67). Linear tooth movement by frontal resorption can only commence if the necrotic patches in the PDL are eliminated and the PDL is repopulated with cells.

IL-1, which binds to IL-1 receptors, directly affects bone metabolism by inducing osteoclast activity (68). In this context, it is worth mentioning that IL-1 gene cluster polymorphisms are associated with the tooth movement velocity, and that naturally occurring IL-1 receptor antagonists diminish the number of TRAP-positive osteoclasts, thus reducing tooth movement (69, 70). Clinicians should be aware that numerous IL-1 and IL-6 gene variants increase the risk of external apical root resorption during OTM, demonstrating the strong impact of inflammatory mediators on the activity of osteoclasts and odontoclasts/cementoblasts, both of which are derived from circulating precursor cells in the PDL (71). IL concentrations in the GCF vary significantly over time when teeth are moved with constant force. The source of inflammatory mediators during tooth movement may not only be compressed fibroblasts but also bacterial-induced gingivitis, tissue irritations caused by the appliance, or the presence of necrotic tissue regions in the pressure zone, which explains the varying concentrations and inconsistent effects of long-term pain reliever use on OTM rate (36, 65, 72).

Patients taking non-steroidal anti-inflammatory medicines for pain treatment may experience an altered OTM process since an inflammatory reaction appears to be unavoidable. Non-steroidal anti-inflammatory drugs suppress cyclooxygenase, the enzyme responsible for the synthesis of prostaglandins; both components are plentiful in the PDL's resorptive compression zone during OTM. However, in the late stages of OTM with constant forces, inflammation may be regulated to physiological levels, with inflammatory mediators playing a smaller role in signaling. Ibuprofen and loxoprofen are examples of pain medicines that have no effect on tooth mobility (72). Nevertheless, practitioners should evaluate the medication used by their patients, particularly since orthodontic treatment in the adult population has increased dramatically in recent decades.

Given the potential to accelerate OTM by inflammatory mediators, numerous procedures, including corticotomy, piezocision, and micro-osteoperforation, have been used to induce a regional acceleratory phenomenon (73). However, excessive proinflammatory factors, each patient's genetic predisposition (e.g., gene polymorphisms in inflammatory mediators), or the combination of sterile inflammation with a microbe-induced inflammatory reaction, such as in patients with periodontitis, may result in adverse side effects during or after OTM treatment (74, 75). Unwanted tissue lesions related with OTM include external apical root resorption, alveolar bone dehiscence, and gingival recessions (76, 77).

Indeed, various IL-1 and IL-6 polymorphisms have been found that increase the risk of external apical root resorptions during OTM (71, 78). For all of these reasons, OTM is regarded to be safer when proinflammatory cytokines are at physiological levels and the inflammatory pathways are not overactive. Furthermore, increased proinflammatory mediators are not necessary for tooth movement, particularly during the post-lag phase (36, 79).

## 4 Apposition in the tension zone

**Figure 3:**
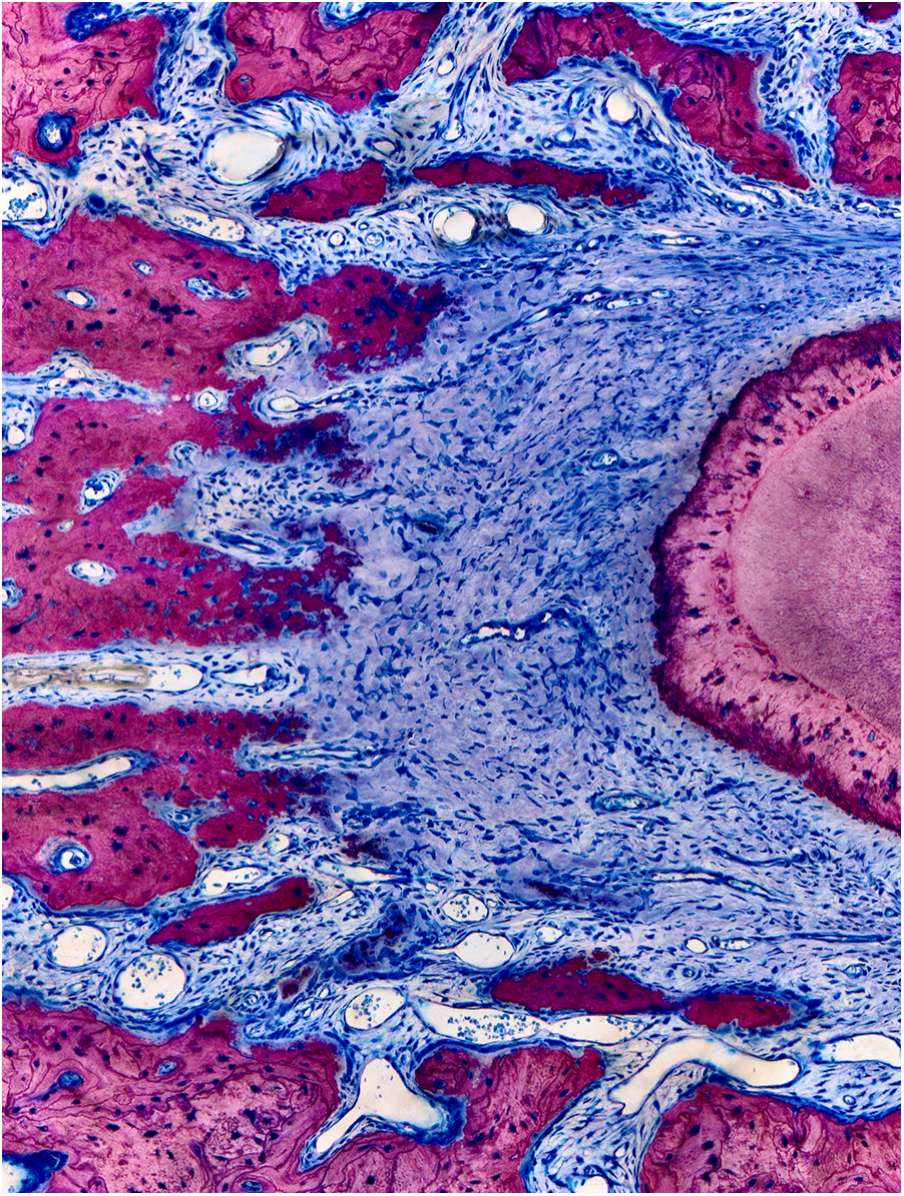
Columnar shaped bone apposition at the periodontal tension zone. The PDL is wider in the tension zone. Osteoblasts (dark blue) are actively depositing osteoid, which is mineralized thereafter (dark pink to light pink) to restore the normal width of the periodontium. This primary bone is characterized by immature bone trabeculae which are aligned along the direction of traction exerted by the periodontal fibers. Secondary bone can be identified by its osteon-like shell structure and the light pink color (visible on the left corners). Characteristic bone layers of osteons are not visible in fresh bone, indicating it has not undergone remodeling yet (15).

While the alveolar bone in front of the root resorbs, permitting tooth movement, the wider PDL on the opposite (rear) side must be restored to normal by the apposition of new bone (Figure 3). Osteoblasts, which differentiate from mesenchymal stem cells (MSCs), are bone-forming cells that secrete the bone matrix. The commitment of MSCs to differentiate into osteoblasts necessitates the spatiotemporal expression of particular genes. These include the transcription factors Runt-related transcription factor-2 (Runx2) and Osterix. Runx2 is a master regulator of osteoblastic differentiation, as evidenced by the complete lack of osteoblasts in Runt-related transcription factor-2-null mice (80).

Osteoblasts function as mechanosensors, transforming external mechanical inputs into biological signals within the cell. As a result, they can respond to a wide range of external stimuli by producing cytokines and mediators such as OPG, RANKL, and bone morphogenetic proteins (BMPs). Mechanotransduction occurs when extracellular matrix proteins interact with integrin receptors, which are linked to the cell's cytoskeleton via numerous integrin-binding proteins (27, 81, 82). Mechanical stimuli are vital for bone physiology; for instance, mechanical loading is required for mandibular osteoblast survival in order to preserve the mandibular alveolar process (83). While physical exercise has only a slight effect on bone mass, it is well recognized that the reduced gravity force experienced by space travelers causes a decline in bone mineral density (84–86). The most important signaling molecules for osteoblastic activity are Wnt- and BMP-activated signaling cascades.

### 4.1 Wnt signaling

Wnt signaling is a key regulator of osteoblast differentiation. Wnt proteins bind to the Wnt-receptor complex, which consists of a transmembrane G-protein coupled receptor from the Frizzled family and an LRP co-receptor (87) or a Ryk or Ror tyrosine kinase (88). Binding or mechanical stimulation activates intracellular cascades, including the canonical pathway, which stabilizes and translocates β-catenin to the nucleus. β-catenin, a transcriptional coactivator, regulates gene transcription in response to Wnt signaling activation. Wnt signaling can be activated in bone tissue when secreted frizzled-related protein 1, Sclerostin, or Dickkopf-1 are missing or present in low concentrations. Active Wnt signaling increases the number and activity of osteoblasts (89–91) by upregulating the direct target genes *RUNX2* and *OPG* in osteoblasts (92, 93).

**Figure 4:**
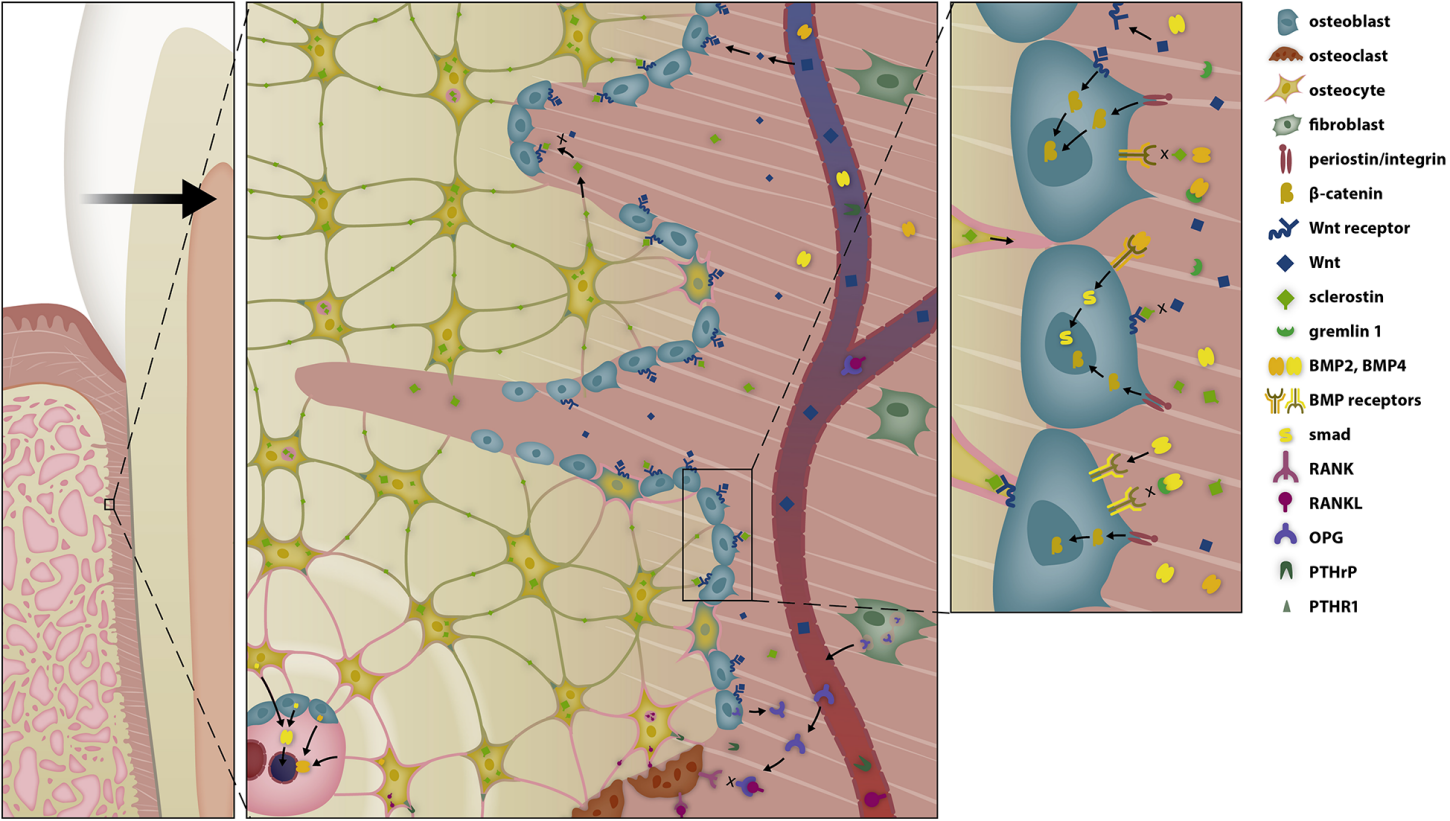
Selective modulation of the BRRS for apposition by activation of osteoblasts in the late phase of OTM. Osteoblasts are stimulated by several pathways to form osteoid. Tension on the extracellular matrix is transduced into intracellular signal cascades via integrins, while Wnt activates the Wnt-receptor complex, both resulting in the migration of β-catenin into the nucleus, where it modifies gene expression. The response is further modulated by bone morphogenetic proteins (BMPs) and BMP antagonists.

In a rat OTM model, orthodontic force application increased Wnt3a, Wnt10b, and β-catenin levels in the tension side of the PDL (94). This result is consistent with increased osteoblast activity on the tension side. During tooth movement, mechanotransduction is assumed to be the primary mechanism of Wnt activation in osteoblasts (Figure 4). Collagen fibers, which are attached to the cell membrane via integrins, are stretched, producing intracellular signals. Wnt and integrin signaling pathways converge to stabilize and translocate β-catenin into the nucleus, promoting bone formation through gene expression (95, 96). The binding of a Wnt cytokine to the Wnt-receptor complex results in the release of intracellular β-catenin (97). For example, exercise training upregulates apelin expression in muscles, which has a paracrine effect on surrounding bones by activating the Wnt pathway in osteoblasts, hence boosting bone formation (98, 99). It is hypothesized that this mechanism shapes the bone's surface. Increased Wnt activation increases OPG release from osteoblasts and reduced osteoclast activation, which slows bone resorption (97). Overactivation of Wnt10b has been linked to delayed incisor eruption, increased mandibular bone, and higher femoral bone mineral density (100).

### 4.2 BMP signaling

As osteoclasts resorb bone, growth factors such as BMPs are released from the mineralized bone matrix, attracting osteoblasts to the site of resorption. BMPs belong to the transforming growth factor-β superfamily and control bone metabolism through both SMAD-dependent and SMAD-independent pathways. Twelve different BMP ligands (101) have been discovered to bind with variable affinity to different BMP receptor complexes. BMPs bind as dimers to their receptors, often in combination with other molecules, eliciting a wide range of responses depending on the BMP ligand–receptor combination. Although not all BMPs are osteogenic effect (102), the deletion of various BMP signaling mediators leads to a bone phenotype during skeletal development or to bone defects later in life (103). For instance, BMP receptor-1B deletion causes temporary and gender-specific osteopenia, suggesting that alternative signaling pathways may compensate for this component in bone formation (104).

BMPs are potent osteogenic mediators that influence stem cell differentiation and bone fraction healing (105, 106). BMP-2 is considered the gold standard for bone regeneration and its use in orthopedics has been encouraged in cases of non-union fracture and spinal fusion (107, 108), as well as for regenerative purposes in dentistry (109). Osteocytes and osteoblasts in Haversian canals express BMP-2, which is induced upon encountering mechanical stress (110). However, mechanical tension not only elevates levels of BMP-2, but also BMP-4 (111). Several BMPs, including BMP-2, BMP-4, BMP-5, BMP-6, BMP-7, and BMP-9 have been shown to improve bone formation and/or regeneration (106). One way that BMP signaling may enhance bone growth is by boosting Wnt signaling. Indeed, most BMPs, including BMP-4 and BMP-7, and to a lesser extent, BMP-2, BMP-3, BMP-5, and BMP-6, increase Wnt expression (105). These findings indicate that some BMPs can promote bone formation through β-catenin translocation, analogous to integrin-dependent mechanotransduction or Wnt activation by muscle activity via apelin.

BMPs could possibly be implicated in tooth movement (112). BMP-2 levels in the tension sides of the PDL rise in response to the tension strain (113). BMP-2 stimulates mineralization in pre-osteoblastic cells through an autocrine loop, resulting in a synergistic effect with Wnt activation (47, 114). This autocrine BMP activation and mechanotransduction via integrins may cause an increase in stem cells and Wnt activation in the tension zones during the first 10 days of OTM (112). Maintaining optimal orthodontic force is critical because the amount of compressive strain influences BMP-2 induction (2).

BMP antagonists bind to extracellular BMPs, inhibiting the activation of type I and type II receptors to form a trans-phosphorylated receptor complex (115). Various BMP antagonists have been described in developmental gene expression and in physiologic BRRS, such as Noggin, Chordin, Crossveinless-2, Twisted gastrulation, Gremlin-1, Gremlin-2, Gremlin-3, NBL1, Cerberus, Sclerostin, and sclerostin domain containing protein-1 (116, 117). The regulation of the expression of Gremlin-1 could be particularly important during tooth movement, as it is much higher than other BMP antagonists in the gingiva and the periodontium (30).

## 5 Cortical bone during tooth movement

**Figure 5:**
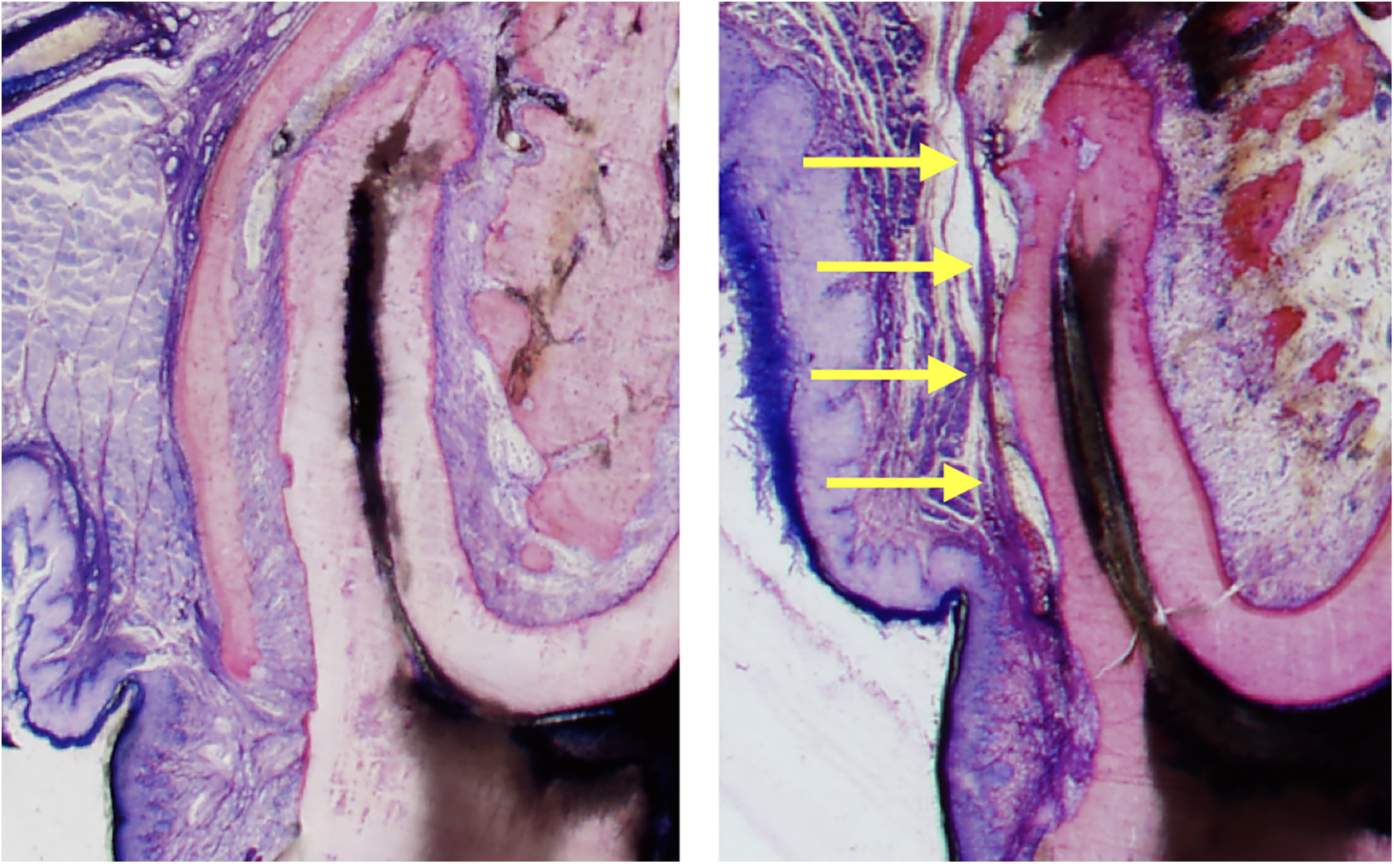
Sequelae of tooth movement far beyond the periosteal surface. The left histomicrograph presents a mesial root cross section of the third molar obtained from a control rat, representing normal alveolar bone and periosteum covering the roots. The right histomicrograph displays a mesial root cross section of the third molar obtained from a rat in the experimental group. The root was displaced (to the left side) in an extreme expansive movement beyond the initial alveolar surface, causing complete bone dehiscence and a fusion of the periosteum with the PDL (yellow arrows) (15).

OTM requires the balanced resorption and deposition of alveolar bone at the PDL compression and tension sides, respectively. However, the success of OTM is determined not only by the amount, duration, and frequency of force application, but also by anatomical boundaries that must be respected. Indeed, there is universal agreement that the thickness of the cortical plate is a clear limit for OTM. Moving the teeth towards the periosteum, or even outside the labial or lingual alveolar plate increases the risk of bone thinning and alveolar bone abnormalities, such as bone dehiscence (Figure 5). In rats, the alveolar bone plate has limited adaptability, accounting for less than 20% of the tooth diameter. This would be equivalent to one millimeter at the cemento–enamel interface in humans, however some inter-individual variation is to be expected (15).

During OTM, mechanosensitive cells such as PDL fibroblasts, osteoblasts, and osteocytes regulate the BRRS by transducing external input into the cell and activating signaling pathways, resulting in specific gene expression modification. Wnt molecules promote bone formation through the Wnt/β-catenin signaling pathway, increasing the number of osteoblasts per surface and resulting in increased bone mineral density (97, 100).

### 5.1 Sclerostin

Under normal settings, osteocytes produce large amounts of sclerostin, an extracellular glycoprotein that prevents excessive bone apposition while allowing for normal bone remodeling (39). Sclerostin, a competitive Wnt antagonist, inhibits ligand binding to the Wnt co-receptor LRP5/6 (97, 117, 118). As a result, sclerostin suppresses replication and differentiation of pre-osteoblasts into osteoblasts while promoting osteoblast apoptosis (39, 119, 120). Hence, elevated concentrations of sclerostin around osteocytes restrict excessive new bone formation. The timing of sclerostin production is critical for differentiating osteocytes from osteoblasts in the apposition area of a bone multicellular units (BMUs) (120). In this region, single osteoblasts differentiate into osteocytes, which are then enclosed within the circular layers of the osteon (119). Differentiated osteocytes on the other hand, maintain lengthy extensions to surrounding osteocytes, establishing a canalicular network that allows for signal exchange (120). Osteocytes along the periosteal and endosteal surfaces communicate directly with osteoblasts, blood vessels, stem cells, and bone marrow cells through continuous channels and by secreting cytokines or macrovesicles (121).

### 5.2 Sclerostin depleted areas facilitate Wnt-activation

**Figure 6:**
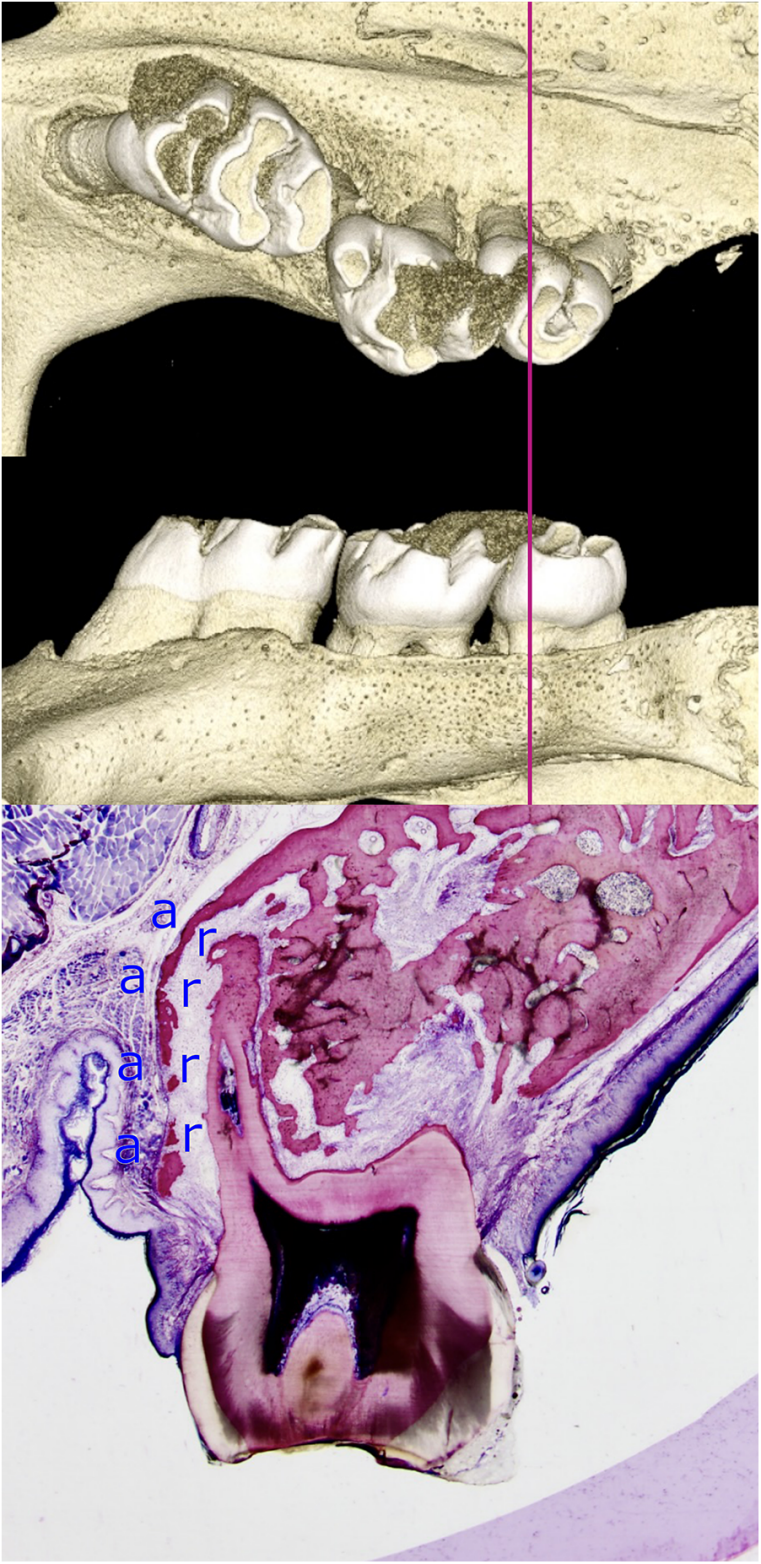
Limited adaptability of the alveolar bone when a root is moved towards the periodontal surface. The histomicrograph of a section through the mesial root of a rat's third molar, shifted towards the periosteum, is shown alongside three-dimensional reconstructions of the micro-computed tomography data. The capacity of bone to adapt is limited during the process of tooth movement towards the periosteum, a finding that has significant implications for the selection of clinical strategy during orthodontic treatment planning. The perforated, thinned alveolar wall is indicative of intense remodeling processes. Osteoblasts located in the periosteum are responsible for bone formation (a), while osteoclasts situated in the PDL facilitate bone resorption (r). The adaptability of bone varies significantly among individuals and is estimated to be less than one millimeter in humans (15).

**Figure 7:**
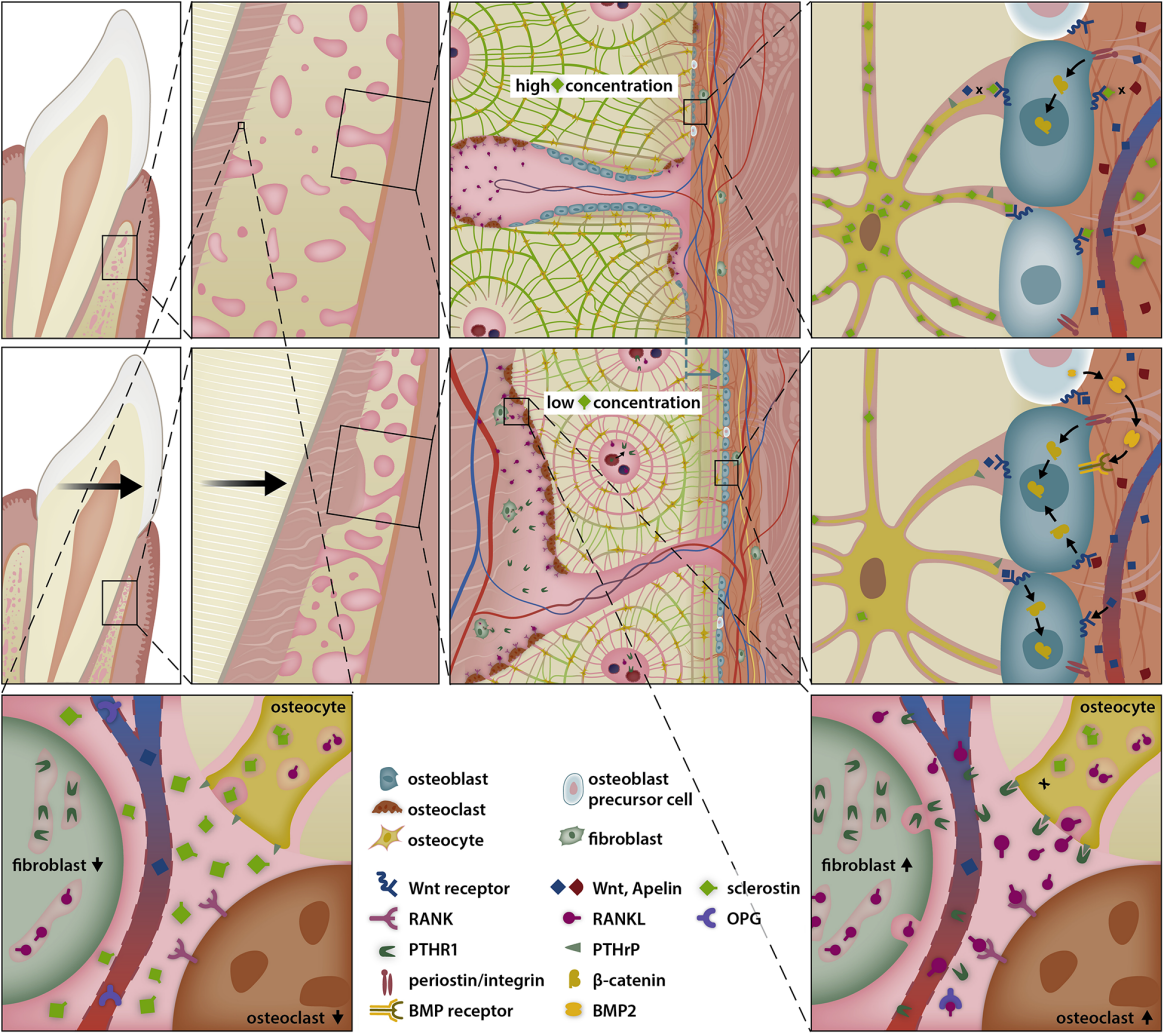
Sclerostin-depleted areas stimulate periostal bone apposition allowing simultanous presence of resorption ahead of the root and apposition at the alveolar surface. Compressed periodontal fibroblasts secrete PTHrP, which diffuses over Haversian canals and activates neighboring osteocytes, thereby favouring bone resorption in the pressure zone of the periodontal ligament. However, PTHrP also reduces the secretion of sclerostin by osteocytes via interaction with the PTHrP receptor type 1 (PTHR1). The suppression of osteocyte sclerostin secretion, in conjunction with the shrinkage of the periosteal bone plate, creates a sclerostin depleted area, where the Wnt-receptor complex and osteoblast precursor cells are less inhibited, thereby promoting bone apposition at the periosteal surface.

When teeth are shifted faciolingually towards the periosteum, the alveolar bone's ability to adjust is limited. Alveolar bone dehiscence is correlated to the amount of movement beyond the alveolar surface and is not modulated by the magnitude of the orthodontic force, as long as the force is applied within a physiological range (15). OTM-mediated pressure zones towards the alveolar bone plate result in bone apposition at the periosteum and simultaneous resorption ahead of the root (Figure 6). β-Catenin-positive cells are primarily observed in tension zones with high bone apposition, but also on the periosteal surface (112). When a root is moved close to the alveolar surface, bone mass and the number of osteocytes decrease, resulting in lower levels of sclerostin near the periosteal surface. Subsequently, PTHrP secreted by PDL fibroblasts binds to osteocytes, further inhibiting sclerostin secretion (39). This creates a sclerostin-depleted region at the alveolar surface where Wnt receptors are unblocked, leading to increased activation via the canonical route with stable and nuclear β-catenin. As a result, osteoblasts are activated, and multipotent precursor cells in the periosteum start to proliferate and differentiate into osteoblasts. Therefore, bone apposition is enhanced at the periosteal surface when sclerostin levels are low (Figure 7). Furthermore, low levels of sclerostin, an antagonist of BMP-6 and BMP-7 (115), promote the activation of the canonical BMP/Smad pathway, which may be another relevant signal cascade that leads to limited bone apposition at the alveolar surface.

### 5.3 Medication affecting periosteal bone-forming

BRRS and OTM are influenced by a variety of pharmacological substances (8, 9, 122). The findings presented herein provide novel therapeutic avenues for minimizing the likelihood of acquiring OTM-related hard tissue defects. Reducing sclerostin concentrations with an anti-sclerostin antibody or anti-miR-19a/b medication enhances bone mineral density and content, which, when paired with bone-loading activities, can result in increased cortical bone thickness (123–125). The combination of anti-osteoporosis drugs such as sclerostin inhibiting antibodies and periodontal PTHrP may have a synergistic effect, potentiating bone growth. This could be a potential method for promoting production of new bone during tooth movement and lowering the risk of periodontal sequelae through medication.

## 6 Discussion

The PDL and bone tissue are very responsive to mechanical stimulation. External force can be perceived by PDL fibroblasts, osteoblasts, osteocytes, and osteoclasts, altering their differentiation status and activity, and thus the entire local microenvironment. Indeed, the tissue remodeling required for OTM is a complex yet precise regulatory mechanism that involves the environment, neighboring cells, and mechanical sensors (e.g., integrins, Yes-associated protein/transcriptional coactivator with PDZ-binding motif, cytoskeleton, Smad signaling, Wnt signaling) within a single cell (126). However, OTM is more than just a periodontal response to external stresses. Tooth movement is the consequence of a complex network of highly coordinated and interrelated biological and mechanical processes involving numerous variables. Clinical factors include the amplitude of orthodontic force, the speed of tooth movement, the direction (mesio-distal, facio-oral, intrusion vs. extrusion) and amount of movement, and the patient's age. Adults have less elastic and flexible alveolar bone (127), fewer progenitor cells and PDL fibroblasts, and a slower bone turnover rate than younger individuals (128). Furthermore, adult patients seeking orthodontic treatment may already have modest periodontal issues, which can jeopardize the effectiveness of OTM (74, 75). A better understanding of BRRS and inflammation is essential to mitigate potential adverse consequences during facio-oral OTM. BRRS is regulated during OTM to cause local resorption ahead of the root or apposition, restoring the usual width of the PDL. OTM has the potential to influence subperiosteal bone biology. Subperiosteal bone apposition occurs when the root moves against the periosteum. The extent of this apposition varies greatly between individuals.

Conflicting findings have been published regarding whether micro-osteoperforation or corticotomies accelerate tooth movement by increasing bone resorption at pressure zones or not (129). The rapid acceleratory phenomenon (RAP) is characterized by increased vasodilation, enhanced capillary permeability, and elevated levels of soluble inflammatory mediators, and it occurs during wound healing following a surgical procedure (130, 131). Already, mobilizing a mucoperiosteal flap is enough to cause a RAP through wound healing (130, 131). Wound healing involves inflammatory cells including activated macrophages, neutrophils, T-lymphocytes as well as proinflammatory mediators like IL-1, IL-6, IL-8, tumor necrosis factor-α, prostaglandins and interferon-c, which promote bone resorption during tooth movement (131). But in addition to their potential to speed up tooth movement, surgical adjunctive interventions also carry a risk of adverse effects like loss of tooth vitality, bacteremia or, via increased tissue inflammation, periodontal issues, severe root resorption, or cortical bone dehiscence (129, 132–134).

Addressing orthodontic discrepancies or space deficiencies that exceed the adaptability of the BRRS requires clinical interventions such as growth modification, space creation through intermaxillary expansion, narrowing of teeth by grinding (interproximal enamel reduction), distalization and/or extractions of permanent teeth, controlled tooth movements against extraoral or skeletal anchorages, or orthognathic surgery (135–141). Distracting parts of the cortical alveolar bone wall during tooth movement and bone grafting has been proposed as a further clinical approach to enhance the alveolar limits and decrease treatment time (142, 143). The impact of corticotomies and micro-osteoperforations lateral to the roots on accelerating tooth movement and reducing or repairing bone dehiscence, when facio-oral tooth movements are planned, remains without supporting evidence (133, 129, 134).

Being mindful of the duration of orthodontic therapy is an important consideration, especially in older patients. It is believed that prolonged treatment periods may increase the risk of acquiring caries, external root resorption, and gingival inflammation, primarily due to poor oral hygiene. As a result, non-invasive procedures to accelerate tooth movement without jeopardizing the structure of the teeth or the periodontal tissues are gaining popularity. The application of low-intensity pulsed ultrasound (LIPUS), photobiomodulation (PBM) with low-level laser therapy (LLLT), and vibration has been proposed as less invasive methods to positively influence BRRS during orthodontic tooth movement:

LIPUS is thought to effect bone remodeling, and thus OTM, by transmitting external energy waves to periodontal tissues. Periodontal cells sense these micromechanical stresses and convert them into biological signals via mechanotransduction processes (e.g., the integrin/mitogen-activated protein kinase pathway). This causes cellular alterations, which have been shown to accelerate OTM in mandibular organ cultures and animal models by increasing alveolar bone remodeling (144–146). Additionally, LIPUS was demonstrated to reduce orthodontically-induced root resorptions (147). However, the precise cell-specific molecular mechanisms leading to these favorable effects of LIPUS on OTM remain speculative. It appears that LIPUS activates mechanotransduction pathways in osteoclasts and osteoblasts (148), which stimulates RANKL expression in osteoclasts (149) and the levels of RUNX2, OPG, and ALP in osteoblasts (150–152). The favorable result of LIPUS treatment on the appearance of root resorptions has been attributed to its suppressive effect on cementoclast activation and signaling modifications that lead to enhanced tissue regeneration (151, 153, 154). However, it should be emphasized that there are many contradictory findings in the literature, and several studies could not disclose any beneficial effects of LIPUS on the rate of OTM and/or prevention of unwanted side-effects (155–157). In addition, a proper comparison of all the outcomes is not possible because multiple LIPUS techniques and strategies (e.g., timing, frequency, energy) were applied (158). At that point, larger and higher-quality randomized-controlled trials with larger sample sizes, standardized methodology, and optimal treatment and control groups will be required to validate the efficacy of LIPUS on OTM (159–161).

PBM with LLLT is another non-invasive method for speeding OTM. PBM has been found to improve bone remodeling, alter inflammatory responses, stimulate cellular activity, and regulate gene expression. All of these mechanisms combined may result in faster and more efficient tooth movement (162). The primary target of LLLT is mitochondria. Since osteoclasts are multinucleated cells with highly active mitochondria, LLLT is quite effective in them. Essentially, low-level laser light is adsorbed by cytochrome oxidase C, the terminal enzyme of the respiratory chain, and chromophores are excited, which activate signaling pathways and biologically active secondary mediators with varying effects on OTM. These factors include reactive oxygen species (ROS), ATP, and NO (163, 164). Ultimately, these molecules promote bone remodeling by enhancing total cellular activity, resulting in tissue regeneration and accelerating OTM. It is thought that LLLT boosts the quantity and activity of osteoclasts and osteoblasts in treated areas (165). Furthermore, laser irradiation stimulates the release of pro-inflammatory molecules (e.g., IL-1b, IL-6, TNFa) in the periodontium, and more osteoblasts express greater levels of RANKL, resulting in the simulation of osteoclastogenesis via the conventional RANKL/RANK/OPG system (164, 166, 167). This effect is sustained by increased levels of fibroblast growth factor 2 (bFGF) as a result of LLLT (168, 169). BFGF enhances synthesis of extracellular matrix proteins such as Fibronectin and Collagen I (170), allowing for faster tooth movement by maintaining the PDL matrix (171). However, the effectiveness of PBM with LLLT on OTM is still uncertain since there is a substantial paucity of high-quality clinical trials and a comprehensive assessment of the many outcomes of the research is impossible due to lack of standardization and completely heterogeneous set-ups (172–174).

Finally, vibration produces alternate stimuli that might improve the pace of OTM through increased bone remodeling (175). Osteocytes sense vibration forces and respond by upregulating NF-kB signaling leading to higher levels of RANKL and TGFb1, which triggers TGFb signaling (176, 177). Therefore, it is thought that vibration activates the NF-κB-TGF-β1-RANKL axis, leading to increased osteoclastogenesis and faster OTM (178). This holds especially true under continuous static force (179). RANKL may potentially be activated by increased prostaglandin E2 secretion by stress-sensing PDL cells as well (180). Although there are numerous studies in the literature that focus on increasing OTM rate by vibration, the results are often equivocal. This makes it difficult to accurately assess the influence of vibration on OTM speed and there is not a standardized protocol available so far.

Collectively, all of these non-invasive adjuvant approaches aimed at accelerating orthodontic tooth movement may be promising, but they are currently quite diverse, and inconsistent clinically meaningful outcomes have been demonstrated with a low level of evidence (156, 157, 173, 174). Short-duration stimuli are unlikely to initiate or alter tooth movement. The BRRS is highly responsive to long-duration, very small forces, such as tongue or cheek forces, but not to short-duration stimuli, such as masticatory forces. It is critical to consider not just the speed of tooth movement but also to search for biochemical changes in BRRS when using vibration, light or ultrasound. Study results are only meaningful if side effects such as bone dehiscences of the alveolar wall or root resorption are considered in addition to tooth movement velocity.

When dentoalveolar compensation for discrepancies or complex therapies are planned, it is imperative to consider modulating the BRRS to reduce adverse effects, such as bone dehiscences. A less invasive and more efficient method of decreasing adverse effects is to pharmacologically modulate the BRRS by locally administering sclerostin-inhibiting antibodies and periodontal PTHrP. This approach, when combined with bone grafting, holds promise in minimizing the invasiveness and complexity of other therapeutical strategies while improving normal adaptability and alveolar limitations, especially in cases with naturally thin periodontal tissues.

**Figure 8:**
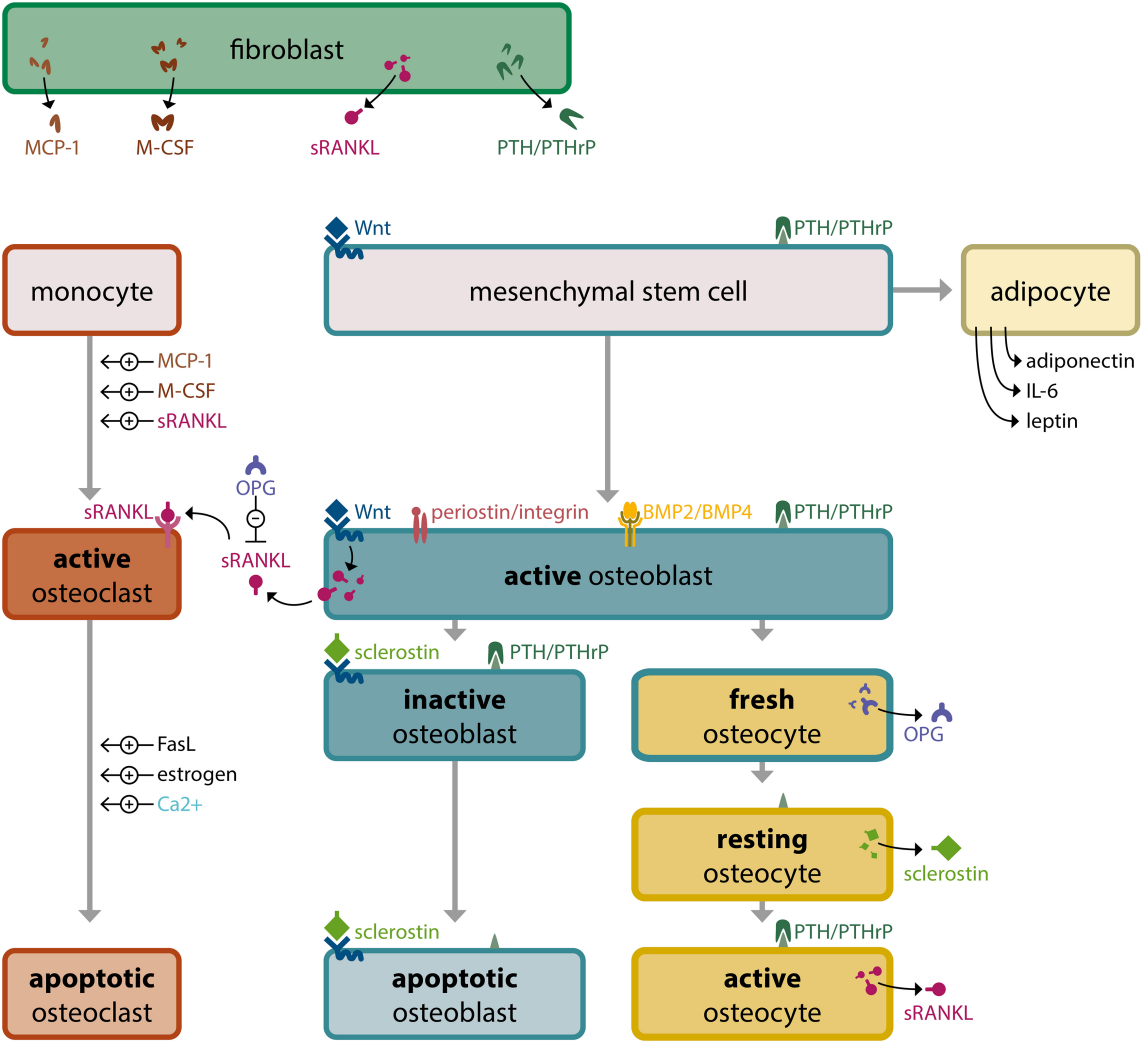
Bone tissue, modulated by the bone remodeling regulatory system (BRRS). The Bone Remodeling Regulatory System (BRRS) provides complex regulation of calcium homeostasis, genetic skeletal development (intramembranous ossification, tooth eruption) and functional needs for adaptation to environmental changes (skeletal function, tooth displacement). Feedback mechanisms such as the Ca-sensitive apoptosis of osteoclasts and the interaction of osteoblasts, osteocytes and osteoclasts are important for an optimal bone shape, structure and quantity. The most important mediators in BRRS and their source cells are: PTH (parathyroid glands), locally produced PTHrP (chondrocytes, fibroblasts), FGF23 (osteocytes), sRANKL (osteocytes, osteoblasts, fibroblasts), Sclerostin (resting osteocytes), OPG (fresh osteocytes, osteoblasts), mechanotransduction over periostin/integrin (extracellular matrix), Wnt (muscle cells, fibroblasts), BMPs (kidney, osteoblasts, osteocytes, chondrocytes), MCP-1 (fibroblasts), M-CSF (fibroblasts), adiponectin (adipocytes), IL-6 (monocytes, macrophages, adipocytes), leptin (adipocytes), FasL (T lymphocytes, macrophages), estrogen (ovaries, mesenchymal cells of adipose tissue including that of the breast, osteoblasts, chondrocytes, vascular endothelium and aortic smooth muscle cells), calcium (bone resorption and serum), phosphorus (bone resorption and serum).

The novel biochemical theory of modulating BRRS via tooth movement offers new clinical options for treating osteoporosis, including the use of sclerostin-inhibiting antibodies and intermittent periodontal PTHrP to promote bone apposition (Figure 8). Anti-sclerostin antibodies or miR-19a/b antagonists given locally in conjunction with PTHrP are intriguing future possibilities for lowering the risk of bone dehiscence during OTM in individuals with poor alveolar bone thickness, which is frequently observed in adults.
